# Molecular Biomarkers for the Diagnosis, Prognosis, and Pharmacodynamics of Spinal Muscular Atrophy

**DOI:** 10.3390/jcm12155060

**Published:** 2023-08-01

**Authors:** Marija Babić, Maria Banović, Ivana Berečić, Tea Banić, Mirjana Babić Leko, Monika Ulamec, Alisa Junaković, Janja Kopić, Jadranka Sertić, Nina Barišić, Goran Šimić

**Affiliations:** 1 Department of Neuroscience, Croatian Institute for Brain Research, University of Zagreb School of Medicine, 10000 Zagreb, Croatia; 2 Department of Pathology, University Clinical Hospital Sestre Milosrdnice Zagreb, 10000 Zagreb, Croatia; 3 Department of Pathology, University of Zagreb School of Medicine, 10000 Zagreb, Croatia; 4 Department of Medical Chemistry and Biochemistry, University of Zagreb School of Medicine, 10000 Zagreb, Croatia; 5 Department of Laboratory Diagnostics, University Hospital Centre Zagreb, 10000 Zagreb, Croatia; 6 Department of Pediatrics, University Hospital Centre Zagreb, 10000 Zagreb, Croatia

**Keywords:** spinal muscular atrophy, survival motor neuron 1 protein, pharmacological biomarkers, prognosis, nusinersen

## Abstract

Spinal muscular atrophy (SMA) is a progressive degenerative illness that affects 1 in every 6 to 11,000 live births. This autosomal recessive disorder is caused by homozygous deletion or mutation of the *SMN1* gene (survival motor neuron). As a backup, the *SMN1* gene has the *SMN2* gene, which produces only 10% of the functional SMN protein. Nusinersen and risdiplam, the first FDA-approved medications, act as *SMN2* pre-mRNA splicing modifiers and enhance the quantity of SMN protein produced by this gene. The emergence of new therapies for SMA has increased the demand for good prognostic and pharmacodynamic (response) biomarkers in SMA. This article discusses current molecular diagnostic, prognostic, and pharmacodynamic biomarkers that could be assessed in SMA patients’ body fluids. Although various proteomic, genetic, and epigenetic biomarkers have been explored in SMA patients, more research is needed to uncover new prognostic and pharmacodynamic biomarkers (or a combination of biomarkers).

## 1. Spinal Muscular Atrophy

Spinal muscular atrophy (SMA) is a progressive degenerative disease characterized by muscle weakness and atrophy. It is an autosomal recessive disorder, affecting 1 in 6–11,000 live births [1]. SMA is caused by a mutation or homozygous deletion of the *SMN1* gene (survival motor neuron) found on chromosome 5q11.2–q13.3. In 95% of individuals, SMA is caused by the homozygous deletion of exon 7 in the *SMN1* gene, while homozygous point mutation or heterozygous mutation (deletion and point mutation) is detected in less than 2% of patients [2]. Multiple cellular processes, including RNA metabolism, ribonucleoprotein assembly, trafficking, signal transduction, and actin dynamics [3,4], are dependent on the SMN protein, which is essential for the normal functioning of the cell. Based on the age at which the first signs and symptoms of the disease appear, SMA can be divided into five subtypes. SMA type 1 (Werdnig–Hoffmann disease) is the most frequent form of SMA, with an incidence of 50−60% [5]. It presents in the first 6 months of infancy with generalized hypotonia, muscle weakness, poor motor abilities, areflexia, swallowing, feeding, and breathing difficulties, respiratory failure, and premature death before the age of 2 years. In the natural course of the disease, only 8% survive until the age of 20 months, and survivors never sit unassisted. In SMA type 2, the first signs appear at the age of 7 to 18 months. These children never walk unsupported and suffer from progressive scoliosis and persistent respiratory insufficiency that may limit their life expectancy. Signs of progressive muscle weakness in SMA type 3 (Kugelberg-Welander disease) develop after 18 months. SMA type 3 is separated into two subgroups: SMA3a manifests before the third year of life, whereas SMA3b develops after the age of 3 years. SMA type 4 is the least frequent and is characterized by mild hypotonia and the slow progression of proximal muscle weakness, with the onset occurring in the second or third decade of life. SMA type 0 manifests with fetal hypomotility or akinesia prenatally, and these children most often die within the first several days of life due to severe generalized hypotonia, muscle weakness, and respiratory and cardiovascular failure [5].

According to recommendations published in 2018 [6], all patients with SMA should undergo a neurological examination every six months that includes a functional, scale-based assessment of motor function, evaluation of respiratory functions, ventilatory and nutritional support, and assessment of somatic development (weight, length, and head circumference). The Expert Consensus Agreement for SMA, CareMArtCARE—a platform used to collect real-life outcome data of patients with spinal muscular atrophy—is based on the use of several functional motor scales for patients with SMA related to age and the results of measurement scores. Scales are used as clinical tools and as functional outcome measures for clinical trials as well. The choice of scale is predominantly determined by the type of SMA and the patient’s age. The Children’s Hospital of Philadelphia Infant Test of Neuromuscular Disorders (CHOP-INTEND) motor skills evaluation scale is used in children under 2 years of age and in patients without the ability to sit independently older than 2 years of age. This 16-item scale evaluates limb flexion and extension, head stabilization, spontaneous movements, and hand grip strength while holding a toy or the examiner’s finger [7]. On the scale, each item is scored from 0 to 4, and the highest possible score is 64. The examination provides physicians with information regarding whether a patient’s abilities correspond to her/his clinical condition [7]. The Bayley Scales of Infant and Toddler Development, third edition (BSID-III), is used for the assessment of developmental functioning in infants and toddlers and involves five domains: cognition, language, social-emotional, and motor and adaptive behavior. The Hammersmith Infant Neurological Examination Section (HINE) is divided into three sections, encompassing 37 items and enabling the quantification of neurologic function assessment in infants. HINE-2 (score 0–26) enables the assessment of motor function development based on the achievement of eight motor milestones: voluntary grasp, kicking, head control, rolling, sitting, crawling, standing, and walking [5].

The Hammersmith Functional Motor Scale (HFMS) is intended for patients with SMA types 2 and 3, as well as those older than 2 years. It consists of 20 items that are scored from 0 to 2 points, depending on whether the patient performs them without assistance, with assistance from others, or is unable to perform them [8]. The maximum number of points is 40. The Hammersmith Functional Motor Scale Expanded (HFMSE) was devised as a modification of the HFMS [9] to adapt the HFMS to patients with SMA types 2 and 3 older than 2 years with the ability to sit, as well as to patients with a CHOP-INTEND score >50. It consists of 33 items that are scored from 0 to 2 points, and the maximum possible score is 66 [9]. To evaluate the motor function of the upper limbs, the Revised Upper Limb Module (RULM) (score 0–37) is used in patients older than 2 years of age with the ability to sit in a wheelchair. In ambulant patients older than 3 years of age, the 6- or 2-Minute-Walk Test (6MWT) is performed and additionally used as an endurance test [5].

Additional scales used for the assessment of motor functions are muscle function measurement scales (MFM-20), the Medical Research Council (MRC) test scale, and the revised Amyotrophic Lateral Sclerosis (ALS) functional rating scale [5].

## 2. Spinal Muscular Atrophy Genetics

SMA is caused by the homozygous loss of the *SMN* gene, which is located on the long arm of the fifth chromosome (5q13) [10]. During the course of evolution, the *SMN* gene duplicated, resulting in two distinct forms: the *SMN1* gene (telomeric copy) and the *SMN2* gene (centromeric copy). The only difference between the *SMN2* gene and the *SMN1* gene is the substitution of cytosine with thymine in exon 7 (840C < T) [11]. This substitution is sufficient to mutate the *SMN2* gene because exon 7 of the *SMN2* gene is rendered inactive. In healthy individuals, the *SMN1* gene produces the SMN protein; therefore, the *SMN2* gene is inactive. The homozygous deletion of exon 7 of the *SMN1* gene causes SMA in approximately 95% of cases; thus, it is appropriately referred to as a disease-determining gene [12]. Nonetheless, additional genes also play a role in SMA. The onset of the first symptoms, the type of SMA, and the severity of the clinical picture are determined by additional genes predominantly located in the 5q13 region. These genes are referred to as disease-modifying genes, with *SMN2* being the most important. Even though the *SMN2* gene is silenced and generates only 10% of the functional SMN protein, this gene is essential for survival in SMA patients [2]. The number of *SMN2* gene copies differs from person to person. A higher number of copies of the *SMN2* gene is associated with a milder clinical phenotype of SMA [13]. The NLR family apoptosis inhibitory protein/*BIRC1* gene (*NAIP* gene) is included among disease-modifying genes. The deletion of the *NAIP* gene has been linked to a more severe form of SMA [14,15]. The *SMN2* c.859 gene variant, which increases the likelihood of exon 7 inclusion and SMN protein production, is linked to an attenuated disease course [16]. The case of asymptomatic females with homozygous deletion of the *SMN1* gene is intriguing. Oprea et al. discovered a significantly higher expression of plastin 3 (encoded by the *PLS3* gene on the X chromosome) in these females. Experiments on model organisms (mouse embryos and zebrafish) revealed that increased expression of *PLS3* (which is essential for axonogenesis) protects against SMA by maintaining the length of axons (which is crucial because, in SMA, axon formation is impaired due to a mutation in the *SMN1* gene) [17]. A few years later, the same research group discovered the *CORO1C* gene (coronin 1C), which also protects SMA patients. Higher *CORO1C* expression maintains endocytosis in cells lacking the *SMN1* gene, which is also significant because individuals with SMA exhibit a dramatic decrease in endocytosis [18].

## 3. Spinal Muscular Atrophy Pathogenesis

**Figure 1:**
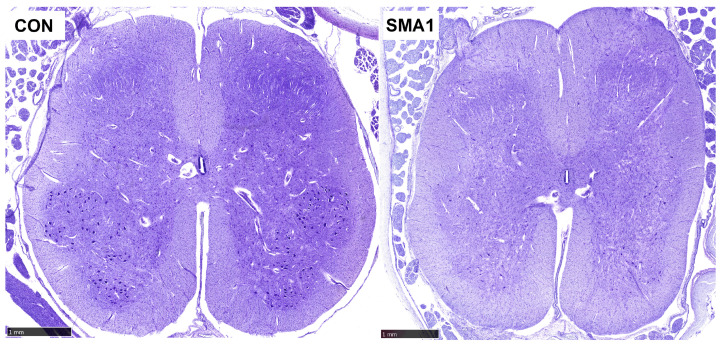
The normal spinal cord of a 4-month-old infant (**left**) and spinal cord of a 5-month-old infant with SMA type 1 due to homozygous deletion of *SMN1* exon 7 (**right**). Both are transverse sections through the cervical part of the spinal cord. SMA1 is characterized by a significant loss of anterior horn a-motoneurons. CON, control; SMA1, spinal muscular atrophy type 1. Cresyl violet stain. The selected sections are part of the Zagreb Collection of human brains at the Croatian Institute for Brain Research, curated by Goran Šimić. Scale bars = 1 mm.

More than 98 percent of SMA patients have a deletion of exon 7 of the *SMN1* gene, and all SMA patients have at least one copy of the *SMN2* gene [10]. Low levels of SMN protein produced by patients with SMA type 0 allow embryonic development but are insufficient to preserve spinal cord motor neurons until the end of pregnancy [10]. Guido Werdnig described pathological changes in SMA in the 19th century, including the loss of neurons in the anterior horn of the spinal cord (mostly α-motoneurons, but also interneurons and γ-motoneurons) (Figure 1), empty spaces in spinal cord tissue where motoneurons had died (“empty cell beds”), bundles of glial cells in the ventral roots of the spinal cord, and heterotopic motoneurons (neurons that do not have synapses and die over time; quickly by apoptosis in the earlier stages of the disease, then slowly by necrosis in the later stages of the disease) [19,20]. Occasionally, SMA can also affect bulbar motoneurons. In addition to the aforementioned pathological processes, numerous other pathogenetic mechanisms have been described over the years in patients with SMA, including (1) aberrant formation of axons and dendrites of motoneurons; (2) impaired formation of synapses between lower and upper motoneurons; (3) abnormal migration of motoneurons in the direction of the ventral root of the spinal cord; (4) abnormal migration of heterotopic motoneurons; (5) neuromuscular junction dysfunction; (6) motoneuron apoptosis; (7) impairment of axonal transport of actin; and, ultimately, (8) death of neurons by apoptosis and necrosis [20,21,22].

**Figure 2:**
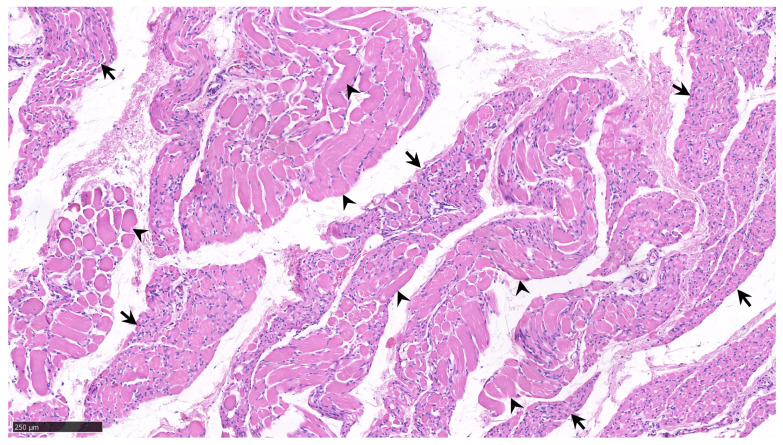
Tissue biopsied from the biceps brachii muscle of a 12-month-old infant with homozygous deletion of *SMN1* exon 7 and only two *SMN2* genes, as well as type 1 spinal muscular atrophy. The arrows show fascicles (groups) of atrophic muscle fibers with pyknotic nuclei interspersed with reinnervated clusters of hypertrophied muscle fibers (arrowheads). There are no signs of severe muscle fiber destruction or phagocytosis. Hematoxylin and eosin stain. The selected section is a part of the Zagreb Collection of human brains at the Croatian Institute for Brain Research, curated by Goran Šimić. Scale bar = 250 μm.

Clinical manifestations of SMA include hypotonia, areflexia, the absence of deep tendon reflexes, weakness in the proximal muscle groups of the trunk, muscle atrophy, and fasciculations of the tongue muscles [20]. Initially, the proximal striated muscles of the extremities become weak; moreover, as the disease progresses, the distal striated muscles and, ultimately, the trunk muscles are also affected (Figure 2). SMA also affects other organs and organ systems, causing changes in the liver, spleen, lungs, heart, and kidneys (primarily in a severe form of the disease with an extremely early onset, type 1a, becoming symptomatic within the first 15 to 30 days of life, and already intrauterine symptomatic type 0).

## 4. Spinal Muscular Atrophy Treatment

Nusinersen is the first FDA-approved treatment for SMA. It is an antisense oligonucleotide that specifically targets the intronic splicing silencing site (ISS-N1) on intron 7 of the *SMN2* gene. ISS-N1 prevents the inclusion of exon 7 of the *SMN2* gene into *SMN2* mRNA; as a result, the transcription and translation of the *SMN2* gene yield only 10% of the functional SMN protein. Nusinersen binds to the ISS-N1 sequence and corrects the previously mentioned excision deficit, thereby enabling the inclusion of exon 7 in *SMN2* mRNA. Thus, the production of full-length SMN protein is increased [23]. Patients receive 5 mL (12 mg) of nusinersen through lumbar puncture via intrathecal administration. Before administration, 5 mL of cerebrospinal fluid (CSF) is withdrawn, and the same volume of dissolved nusinersen is injected in the “opposite” direction. Patients receive four doses of nusinersen over the course of the first three months (0, 14, 28, and 63 days), followed by a single dose every four months [24]. Moreover, there are additional SMA treatment options. Onasemnogen abeparvovec, a gene replacement therapy for the treatment of SMA in infants and toddlers younger than 2 years, was approved by the FDA in 2019. A single intravenous infusion carrying an adenoviral vector (AAV9) containing a functional copy of the *SMN1* gene is administered for approximately one hour [25]. The following year, approval was also granted for risdiplam. This small molecule is a pyridazine derivative that acts similarly to nusinersen, enhancing the incorporation of exon 7 into the mRNA of the *SMN2* gene. This medication is administered orally [26].

## 5. Spinal Muscular Atrophy Molecular Biomarkers

### 5.1. Diagnosis and Prognosis of SMA Using Molecular Biomarkers

Biomarkers are measurable indicators of a specific biological condition. Biomarkers can be measured in body fluids (blood, CSF, and urine), but electrophysiological and neuroimaging techniques are also considered biomarkers [27,28,29]. A strong biomarker has a sensitivity and specificity of at least 85 percent and a correlation with disease progression. Availability, reproducibility, and non-invasiveness are additional substantial characteristics of potential biomarkers [30,31]. Biomarkers can be divided into two categories: biomarkers of disease and exposure. Biomarkers of disease include diagnostic, prognostic, and state biomarkers as well as pharmacodynamic (response) biomarkers, whereas biomarkers of exposure are used to estimate disease risk factors [32,33].

With a growing understanding of the etiology and pathogenesis of SMA and the emergence of new therapeutics, it has become necessary to monitor the progression of the disease and the response to treatment. To establish a diagnosis of SMA, the deletion of the *SMN1* gene and the number of copies of the *SMN2* gene are crucial. The role of *SMN2* as a biological marker is also significant in the natural course of the disease (patients who have not yet begun treatment), as the number of *SMN2* gene copies is a prognostic factor and a modifier of disease severity (that predicts the severity of the disease’s natural progression) [10]. Early SMA onset is associated with a lower *SMN2* copy number, which has an impact on reduced survival, while later SMA onset is associated with more than 2 *SMN2* copies, and motor milestones achieved in the natural course are related to longer survival. The *SMN2* copy number has no impact on functional motor decline or treatment outcome. 

SMN, mRNA, and SMN protein. SMN, mRNA, and SMN protein are produced by transcription and translation of the *SMN2* gene in SMA patients. Different research groups have measured the levels of SMN, mRNA, and SMN protein in the blood and, less frequently, in the CSF [34,35,36] (Table 1). Despite the fact that SMN, mRNA, and protein levels do not change with disease progression, they provide valuable insight into the present disease state [37].

Neurofilaments. Neurofilaments (Nfs) are proteins of the neuronal cytoskeleton that maintain the axon’s structural integrity. Neurofilaments are heteropolymers made up of four subunits: heavy, medium, and light neurofilament chains (NfH, NfM, and NfL; neurofilament heavy, medium, and light chains, respectively), α-internexin (in the central nervous system), and peripherin (in the peripheral nervous system) [38]. There is an increase in the concentration of Nfs in the interstitium, CSF, and peripheral circulation if there is neuronal damage and axon disintegration [39]. Most often, the levels of phosphorylated neurofilament heavy chain (pNfH) and neurofilament light chain (NfL) were measured in SMA patients as being increased or very high, both in pre-symptomatic SMA patients with 2 *SMN2* copies and symptomatic SMA patients [40]. In recent years, NfH and NfL have emerged as promising biomarkers for monitoring SMA progression and as a response to nusinersen therapy in neonates and infants [37]. NfL is substantially elevated in pathological conditions such as multiple sclerosis, Alzheimer’s disease, and ALS [41,42,43].

Creatinine. As a waste product of the creatinine kinase system, creatinine is an indicator of muscle mass [44]. A correlation between serum creatinine and motor functions has been demonstrated [45,46], as well as the former’s potential as a prognostic biomarker of SMA [47]. 

In order to identify new diagnostic and prognostic biomarkers for SMA, studies have analyzed the whole proteome, transcriptome, metabolome, and microRNAome (miRNAome) in the biological fluids of SMA patients (Table 1). 

Whole proteome. Several studies have compared the whole proteome between SMA patients and HCs and correlated significant proteins with scores on scales for the assessment of motor functions. Bianchi et al. analyzed the whole proteome in the CSF of 10 SMA 1 patients and 7 healthy controls (HCs) and observed 39 differentially expressed proteins between SMA patients and HCs (with APOA1, hemoglobin subunit β, hemoglobin subunit α, and transthyretin being the most significant) [48]. In extracellular vesicles released from fibroblasts of one SMA 1, two SMA 2, and three HC subjects, Roberto et al. observed 116 differentially expressed proteins (with IGFBP3, Plastin 3, PTK7, TCP1, FETUA, and FXA being the most significant) [49]. Kobayashi et al. analyzed nearly 1000 plasma proteins in 266 SMA patients and 22 HCs. They even developed a commercial SMA-MAP biomarker panel, including 27 proteins (Table 1) [50]. Another study that analyzed the plasma proteome observed 97 plasma proteins in correlation with the MHFMS score, with TNXB, CILP2, COMP, CLEC3B, ADAMTSL4, THBS4, OMD, LUM, DPP4, PEPD, and CDH13 being the most significantly differentially expressed between SMA patients and HCs [34].

Whole miRNAome. Zaharieva et al. detected 42 differentially expressed miRNAs in the serum of SMA patients compared to HCs [51]. On the other hand, Abiusi et al. detected only an increase in serum miR-181a-5p, miR324-5p, and miR-451a in SMA patients compared to HCs [52].

Whole transcriptome. In a study that conducted a whole blood transcriptomic screen, seven downregulated and three upregulated KEGG pathways were observed, with the most significantly downregulated pathway being “Regulation of Actin Cytoskeleton” [53]. A study that analyzed the transcriptome from fibroblasts using the Gene Expression Plate “Neurodegeneration” observed a decrease in the expression of *SMN1*, *SNCA*, *SV2A*, and *SYN2* mRNA in SMA patients compared to HCs [54].

Whole metabolome. Urinary metabolic profiles successfully differentiated SMA patients from HCs with 81% sensitivity and 98% specificity [55]. Another study showed that even 59 plasma metabolites and 44 urine metabolites correlated with the MHFMS scores [34].

**Table 1:** Prognostic and diagnostic molecular biomarkers of SMA.

Reference	Measured Biomarker	Analyzed Bodily Fluid	Number of Participants	Type of Diagnosis (Number of Patients)	Method Used for Measurement of Biomarkers	Biomarkers in SMA Compared to HCs	Correlation of Biomarkers with Scores on Scales for the Assessment of Motor Functions	Type of Biomarker
[56]	NfL and pNfH	CSF and serum	39	Adult SMA patients (33) HCs (6)	Single molecular array	Unchanged	Negative correlation between serum pNfH concentrations and RULM score, upper and lower extremity strength, and total strength	Pharmacodynamic, prognostic
[57]	IL-1β, IL-4, IL-6, IL-10, IL-17A, IL-17F, IL-21, IL-22, IL-23, IL-31, TNF-α, and IFN-γ	Serum, CSF	44	SMA 1 (4) SMA 2 (13) SMA 3 (16) HCs (11)	Multiplex immunoassay	In serum: ↑IL-1b, ↑IL-4, ↑IL-6, ↑IL-10, ↑IFN-γ, ↑IL-17A, ↑IL-22, ↑IL-23, ↑IL-31, ↑IL-33, ↑TNF-α	No correlation with the HFMSE score	Diagnostic
[51]	Whole miRNAome	Serum	27	SMA 2 (10) SMA 3 (10) HCs (7)	miRNA next-generation sequencing	42 miRNAs differentially expressed; 14 miRNAs upregulated and 28 downregulated		Prognostic
[48]	Whole proteome	CSF	17	SMA 1 (10) HCs (7)	2D-PAGE, MS, WB	↓APOA1 ↑Hemoglobin sub. β ↑Hemoglobin sub. α ↓Transthyretin 2D-PAGE analyses showed 39 protein differences	Not analyzed	Diagnostic
[58]	CHIT1	CSF	109	SMA 1 (7) SMA 2 (33) SMA 3 (39) HCs (30)	ELISA	↑CHIT1	No association with HFMSE and CHOP-INTEND scores	Diagnostic, pharmacodynamic
[55]	Urinary metabolic profiling	Urine	491	Pre-symptomatic (5) SMA 1 (9) SMA 2 (8) SMA 3 (7) DMD (18) HCs (444)	^1^H-NMR-based metabolic profiling combined with sophisticated algorithms based on machine learning	Urinary metabolic profiling	Not analyzed	Diagnostic, prognostic
[49]	Whole proteome	Extracellular vesicles released from fibroblasts	6	SMA 1 (1) SMA 2 (2) HCs (3)	MS, WB	116 differentially expressed proteins (↓94, ↑21) ↑IGFBP3 ↓Plastin 3 ↓PTK7 ↓TCP1 ↑FETUA ↑FXA	Not analyzed	Diagnostic
[39]	NfL in CSF and serum	CSF and serum	115	SMA 1 (4) SMA 2 (7) SMA 3 (3) SMA 2–3 (4) HCs (97), only serum samples	Single-molecule array (SiMoA) assay	↑NfL in serum	A negative correlation of serum NfL with CHOP-INTEND score	Pharmacodynamic, prognostic
[45]	In CSF: NfL, total tau, p-tau_181_, Qalb, OCB, CSF white cells In serum: creatinine	CSF, serum	21	SMA 2 (3) SMA 3 (6) HCs (12)	ELISA	↓Creatinine	A positive relationship between creatinine and HFMSE score and RULM	Diagnostic, prognostic
[52]	Whole miRNAome	Serum	88	SMA 1 (3) SMA 2 (21) SMA 3 (26) SMA 4 (1) HCs (37)	Quantitative real-time PCR	↑miR-181a-5p ↑miR-324-5p ↑miR-451a	Not analyzed	Diagnostic
[59]	*HSPA7* mRNA, HSP70B protein, pNfH protein	Blood and serum	52	Pre-symptomatic (9) SMA 1 (22) SMA 2 (14) SMA 3 (2) HCs (5)	Enzyme-linked lectin assay (for p-NfH), ELISA (for HSP70B), real-time PCR, whole-blood RNA sequencing	↑*HSPA7* mRNA; Positive association of HSP70B protein with pNfH	Not analyzed	Diagnostic, prognostic
[53]	Whole blood transcriptomic screen	Whole blood	65	SMA 3 (31) HCs (34)	L1000 profiling technology and RT-PCR	-Seven downregulated and three upregulated KEGG pathways -Most significantly downregulated pathway “Regulation of Actin Cytoskeleton” (including the expression of key genes in this pathway; *ROCK1*, *RHOA*, and *ACTB*) -↓270 genes, ↑287 genes	Not analyzed	Diagnostic
[60]	NfL	Serum	106	SMA 2 (20) SMA 3 (26) SMA 2/3 (46) HCs (14)	NfL Advantage kit from Quanterix	No difference	Higher levels of NfL were associated with poorer motor performance (as measured by the HFMSE and ALSFRS-R). No correlation between NfL levels and motor scale scores	Pharmacodynamic, prognostic
[47]	Creatinine	Serum	238	SMA 1 (49) SMA 2 (97) SMA 3 (92)	Creatinine data from Project Cure SMA Longitudinal Population Data Repository	Decrease in creatinine levels with disease severity (SMA 3 > SMA 2 > SMA 1)	A positive association with the HFMS score	Diagnostic, prognostic
[61]	pNfH, NfL	CSF	21	SMA 3 (12) HCs (9)	ELISA	No difference	No association with the HFMSE score	Pharmacodynamic, prognostic
[62]	NfL, total tau, GFAP	CSF	23	SMA 1 (12) HCs (11)	ELISA	↑NfL ↑total tau ↑GFAP	A negative correlation of NfL and total tau levels with the CHOP-INTEND score	Prognostic, diagnostic, pharmacodynamic
[63]	pNfH	Plasma	155	SMA 1 (121) HCs (34)	Protein Simple platform	↑pNfH	A negative association between pNfH and the CHOP-INTEND score	Prognostic, diagnostic, pharmacodynamic
[64]	SMN protein	Peripheral blood nuclear cells (CD3^+^, CD19^+^, and CD33^++^ cells)	39	SMA 1 (4) SMA 2 (17) SMA 3 (4) HCs (14)	Imaging flow cytometry	↓SMN protein	A positive correlation between SMN-spot+ cell percentage and the HFMS	Prognostic, diagnostic
[36]	*SMN* mRNA and protein	Blood	53	Infantile-onset SMA (26) HCs (27)	ECL immunoassay, Droplet Digital PCR	↓*SMN* mRNA and protein	A positive association of SMN mRNA with TIMPSI No association with the CHOP-INTEND score	Prognostic, diagnostic
[65]	SMN protein	Exosomes released from fibroblasts and serum	2	SMA 3 (1) HCs (1)	WB	↓SMN protein	Not analyzed	Diagnostic
[66]	*SMN1*, *SMN2-FL*, *SMN2-Δ7*, *GAPDH* and 18S mRNA, SMN protein	PBMCs, skin-derived fibroblasts	443	SMA 1 (18) SMA 2 (60) SMA 3 (52) SMA 4 (5) HCs (293)	ELISA, real-time PCR	↓SMN protein	Correlation between SMN protein concentration in fibroblasts and HFMSE	Prognostic, diagnostic
[67]	SMN protein	Whole blood	52	SMA 1 (5) SMA 2 (22) SMA 3 (22) HCs (3)	ECL immunoassay	↓SMN protein	Not analyzed	Diagnostic
[68]	*SMN* mRNA, SMN protein	Blood	131	SMA 1 (7) SMA 2 (14) SMA 3 (15) HCs (95)	In-house ELISA, multiplex qRT-PCR	A positive association between SMN protein and *SMN* mRNA levels in patients with SMA 1 and SMA 2	Not analyzed	Diagnostic
[69]	SMN protein	Fibroblasts		SMA 1 (1) HC	Imaging flow cytometry technique	↓SMN protein	Not analyzed	Diagnostic
[50]	Analysis of nearly 1000 plasma proteins	Plasma	288	BforSMA samples: SMA 1 (17) SMA 2 (49) SMA 3 (42) HCs (22) PNCRN samples: SMA 1 (35) SMA 2 (66) SMA 3 (57)	Multiplex immunoassay	Development of commercial SMA-MAP biomarker panel (APOB, APCS, ASHG, AXL, CCL2, CD93, CFH, CDH13, CHI3L1, CLEC3B, COMP, CRP, CTSD, DPP4, ENG, ERBB2, FBLN1, IGF1, IGFBP6, LEP, LUM, MB, PEPD, PGF, SPP1, THBS4, TNXB)	A positive association of AXL, CD93, CDH13, COMP, DPP4, LUM, MB, PEPD, SPP1, and THBS4, and negative association of CHI3L1, APCS, and LEP with the MHFMS	Prognostic, diagnostic
[70]	GRP75/Mortalin, Calreticulin	Biopsy of skeletal muscle (*m. quadratus femoris*)	6	SMA 2/3 (3) HCs (3)	Quantitative fluorescent WB	↑Calreticulin	Not analyzed	Prognostic
[71]	*SMN2-FL* transcripts, *SMN-∆D7*, SMN protein	Blood	45	SMA 3	ELISA, real-time PCR	Not analyzed	A positive correlation between *SMN2-FL* transcripts and lower limb Medical Research Council (MRC) score and total MRC score	Prognostic
[34]	Proteome, transcriptome, metabolome	Plasma (proteome, metabolome, amino acids, free fatty acids), urine (metabolome), whole blood (transcriptome)	130	SMA 1 (17) SMA 2 (49) SMA 3 (42) HCs (22)	LC-MALDI-MS/MS Proteomics, Lipid LC/MS Metabolomics, GC/MS Metabolomics, Affymetrix Exon Array	Top 20 markers: (in plasma) TNXB, CILP2, COMP, Glu, CLEC3B, ADAMTSL4, THBS4, OMD, LUM, DPP4, PEPD, C10:0-fatty-acid, CDH13, Asp, Hyp, (in urine) Inositol, Uric acid, Pantothenic acid	97 plasma proteins, 59 plasma metabolites, and 44 urine metabolites correlated with the MHFMS	Prognostic
[72]	SMN protein, SMN transcripts (*SMN2-FL*, *SMN1-FL*, *SMN-∆D7*), *GAPDH* transcript	Blood	130	SMA 1 (17) SMA 2 (49) SMA 3 (42) HCs (22)	ELISA, real-time PCR	↓SMN protein *SMN2-FL* (SMA 3 > SMA 2 > SMA 1) ↑*SMN-∆7* (in SMA 2 and 3) ↓*GAPDH* (in SMA 2 and 3)	No association with the MHFMS	Prognostic, diagnostic
[73]	*SMN-FL*, *SMN-Δ7* mRNA	Blood	61	SMA 2 (42) SMA 3 (19)	Real-time PCR	↓*SMN-FL/SMNΔ7* ratio	Not analyzed	Prognostic
[54]	Transcriptome (Gene Expression Plate “Neurodegeneration”), α-synuclein (SNCA) protein	Fibroblasts, spinal cord mRNA	6	SMA 1 (3) HCs (3)	Real-time PCR, WB	↓*SMN1* mRNA, ↓*SNCA* mRNA, ↓SNCA protein, ↓*SV2A* mRNA and ↓*SYN2* mRNA	Not analyzed	Diagnostic
[74]	SMN protein	PBMCs	7	SMA 1 (1) HCs (6)	ELISA	↓SMN protein	Not analyzed	Diagnostic
[75]	*SMN* mRNA and SMN protein	Blood	86	SMA 1 (6) SMA 2 (9) SMA 3 (14) Carriers (29) HCs (28)	Cell immunoassay, quantitative reverse transcription PCR	↑*SMN-Δ7* mRNA in SMA 2 and 3 patients ↓*SMN-7+* mRNA in SMA 1 patients ↓SMN protein in SMA 1 patients		Diagnostic

(↑) increase, (↓) decrease. The literature search was completed on 2 May 2023. Column “Type of biomarker” was determined based on comments given by the authors of a particular study, but also by observations from the authors of the other studies. 2D-PAGE, two-dimensional SDS-polyacrylamide gel electrophoresis; ALSFRS-R, Amyotrophic Lateral Sclerosis Functional Rating Scale-Revised; CHIT1, chitotriosidase 1; CHOP-INTEND, The Children’s Hospital of Philadelphia Infant Test of Neuromuscular Disorders; CSF, cerebrospinal fluid; DMD, Duchenne muscular dystrophy; ECL, Electrochemiluminescence; ELISA, enzyme-linked immunosorbent assay; FL, Full Length; GFAP, Glial fibrillary acidic protein; HC, healthy control; HFMS, Hammersmith Functional Motor Scale; HFMSE, Hammersmith Functional Motor Scale Expanded; HSPA7, Heat Shock Protein Family A Member 7; HSP70B, heat shock 70 kDa protein 7; IFN, Interferon; IL, interleukin; MHFMS, Modified Hammersmith Functional Motor Scale; miRNA, micro RNA; MS, MALDI-TOF mass spectrometry; NfH, neurofilament heavy chain; NfL, neurofilament light chain; NMR, nuclear magnetic resonance; OCB, oligoclonal immunoglobulin G (IgG) bands; PBMC, peripheral blood mononuclear cells; pNfH, phosphorylated neurofilament heavy chain; pNfL, phosphorylated neurofilament light chain; p-tau_181_, tau protein phosphorylated at threonine 181; Qalb, CSF/serum quotient of albumin; RULM score, Revised Upper Limb Module score; SMA, spinal muscular atrophy; SMN, survival motor neuron; TIMPSI, Test of Infant Motor Performance Screening Items; TNF-α, tumor necrosis factor alpha; WB, Western blot.

Non-molecular biomarkers. In contrast to the previously mentioned molecular biological markers, electrophysiological methods such as compound muscle action potential (CMAP) amplitude, motor unit number estimation (MUNE), and electrical impedance myography (EIM) provide insight into disease severity and progression, enabling the detection of symptomatic patients among pre-symptomatic patients. The aforementioned methods are crucial for detecting symptomatic patients during the so-called pre-symptomatic phases and play an important role as predictive factors in the treatment response [37]. In addition, magnetic resonance imaging (MRI), muscle ultrasound, and, more recently, multispectral optoacoustic tomography, a laser method for determining tissue composition, are used as imaging biological indicators of the symptomatic phase of the disease [37].

### 5.2. Monitoring of Therapeutic Response in SMA Using Molecular Biomarkers

Therapy with nusinersen is a modifying therapy that alters the natural course of the disease: it delays premature mortality, postpones the need for permanent invasive mechanical ventilation, halts progression, stabilizes the disease, and improves the patient’s clinical condition. Measurable molecular markers may contribute to the objectification of the SMA prognosis as well as the prediction and surveillance of the therapeutic effect in SMA [39]. Table 2 demonstrates that NfL and pNfH were used as potential pharmacodynamic (response) biomarkers in the majority of studies that examined the utility of molecular biomarkers for monitoring therapeutic response in SMA. In the majority of studies, a decrease in NfL and pNfH levels was observed following treatment with nusinersen [39,61,62,63,76,77,78,79,80,81], whereas others observed no change in the levels of these biomarkers [45,56,60,82,83,84] (Table 2). 

Tau protein. Total tau (t-tau), an additional marker of neurodegeneration, was evaluated as a potential pharmacodynamic biomarker. Tau proteins stabilize microtubules, and an increase in t-tau levels in CSF is observed when neuronal death occurs [85]. Most studies observed a decrease in t-tau levels in response to nusinersen treatment [62,79,82], whereas others observe no change in the levels of this biomarker [45,84,86] (Table 2). Studies that analyzed phosphorylated tau isoforms after nusinersen treatment observed no change [45] or decrease [84] in phosphorylated tau levels.

S100 calcium-binding protein B (S100B), chitotriosidase 1 (CHIT1), neuron-specific enolase (NSE), and amyloid-β were also investigated as potential pharmacodynamic biomarkers in SMA; however, due to the limited number of studies involving these biomarkers, it is difficult to draw a conclusion regarding the utility of these biomarkers for monitoring the response to nusinersen therapy. 

S100B. S100B protein, which is expressed mainly by astrocytes, belongs to a small dimeric and multigenic calcium-binding family of proteins [87]. An increase in S100B levels was observed in many diseases of the central nervous system. Only two studies analyzed S100B as a pharmacodynamic biomarker, and both of these studies observed no change in S100B levels upon nusinersen treatment [82,86].

CHIT1. CHIT1, which is mainly expressed by activated macrophages in both inflammation and normal conditions [88], was observed to be increased in SMA patients compared to HCs [58]. Few studies have analyzed the pharmacodynamic potential of CHIT1, giving conflicting results. Either an increase [58] or a decrease in CHIT1 levels [89] was observed after the treatment with nusinersen.

Amyloid β. Amyloid β peptide alterations were observed in many neurological disorders. It was also studied as a potential pharmacodynamic biomarker in SMA. Upon nusinersen treatment, there was an increase [90] or no change [84] in amyloid β levels. Other proteins involved in amyloid β metabolism (such as amyloid precursor protein (APP) and BACE-1) were also studied as potential biomarkers in SMA [79,91].

GFAP. Glial fibrillary acidic protein (GFAP) is a type III intermediate filament protein that is expressed mainly by astrocytes [92]. Only two studies have examined GFAP as a pharmacodynamic biomarker in SMA, and both of these studies observed a decrease in GFAP levels following treatment with nusinersen [62,81]. 

Cytokine profile. Several recent studies have examined various inflammatory markers for monitoring therapeutic response in SMA [89,93,94]. Probably due to the analysis of different cytokines and the use of different methodologies, these studies did not yield unifying results. After nusinersen treatment, an increase in G-CSF, IL-8, MCP-1, MIP-1α, and MIP-1β levels and a decrease in IL-1ra, IL-2, IL-4, IL-7, IL-9, IL-12, IL-17, VEGF, eotaxin, and TNF-α levels [93], as well as an increase in IL-10, MCP-1/CCL2 [94], and fractalkine levels [91] and a decrease in IL-8 and IP-10 levels [91], were observed.

Routine CSF parameters. To determine the safety of nusinersen, several studies analyzed the changes in routine CSF parameters following treatment with nusinersen (Table 2). These studies mostly observed an increase in total proteins and the CSF/serum quotient of albumin (Qalb) after the treatment with nusinersen [45,58,95,96,97,98,99] (Table 2). 

Whole proteome, miRNAome, and metabolome. Lastly, some studies analyzed the effects of nusinersen administration on the proteome, miRNAome, and metabolome (Table 2). Magen et al. observed an increase in CSF miR-103b and a decrease in CSF miR-1-3p, miR-133a/b, and miR-206 in nusinersen responders [100], while Welby et al. observed an increase in CSF miR-132, miR-218, miR-9, miR-23a, and miR-146a after treatment with nusinersen [101]. Nusinersen treatment led to an increase in serum miR-335-5p, miR-328-3p, miR-423-3p, miR-142-5p, and a decrease in serum miR-26b-5p [51]. Muscle-specific miRNAs miR-133a, miR-133b, and miR-1 decreased after nusinersen treatment [102]. An analysis of the whole proteome revealed a decrease in CSF Cathepsin D, pNfH and pNfL [77], haptoglobin, and hemoglobin sub. β levels [48], as well as an increase in APOA1 and transthyretin levels [48] and two protein clusters that were identified after nusinersen treatment which differed from the protein clusters at baseline [98].

During nusinersen treatment, various biomarkers were correlated with motor function assessment scale values. Table 2 demonstrates a correlation between certain biomarkers and motor function assessment scale values. There was an inverse correlation between CSF NfL levels and the HFMSE score [79], as well as between CSF [62] and serum [39] NfL levels and the CHOP-INTEND scores. Plasma pNfH levels are negatively correlated with the CHOP-INTEND score [63], serum pNfH levels are negatively correlated with the RULM score [56], and CSF pNfH levels are negatively correlated with the HFMSE and RULM scores [79]. CSF t-tau levels correlated negatively with the CHOP-INTEND score [62,79], whereas serum creatinine levels correlated positively with the HFMSE and RULM scores [45]. Table 2 provides a comprehensive description of additional associations between molecular biomarkers and motor function assessment scale values.

**Table 2:** Review of studies investigating changes in molecular biomarkers in SMA patients treated with nusinersen.

Reference	Measured Biomarker	Analyzed Bodily Fluid	Number of Subjects	Type of Diagnosis (Number of Patients)	The Highest Number of Nusinersen Doses Administered or Treatment Duration	Changes in Biomarker Levels Following Nusinersen Therapy	Correlation between Biomarkers and Motor Function Assessment Scale Values
[93]	IL-1β, IL-1ra, IL-2, IL-4, IL-5, IL-6, IL-7, IL-8, IL-9, IL-10, IL-12 (p70), IL-13, IL-15, IL-17A, IP10, eotaxin, G-CSF, GM-CSF, IFNγ, MCAF/MCP-1, MIP-1α, MIP-1β, RANTES, TNF-α, PDGF-BB, VEGF, FGF-basic	CSF	48	SMA 1 (18) SMA 2 (19) SMA 3 (11) HCs (4)	6 doses (302 days)	In SMA 1 patients: ↓IL-2, ↓IL-4, ↓IL-7, ↓IL-9, ↓IL-12, ↓IL-17, ↓VEGF, ↓eotaxin, ↓TNF-α In SMA 2 patients: ↑G-CSF, ↑IL-8, ↑MCP-1, ↑MIP-1α, ↑MIP-1β In SMA 3 patients: ↓IL-1ra	No association with CHOP-INTEND and HFMSE scores
[89]	CHIT1, IFN-γ, TNF-α	CSF	6	SMA 1 (2) SMA 2 (4)	24 months	↓CHIT1	No association
[94]	IL-1β, IL-3, IL-6, IL-10, IL-12, IL-13, IL-17, IL-18, IFN-γ, BDNF, FAS, VEGF, TNF-α, ANG1, C5a, MCP-1/CCL2	CSF	38	SMA 1 (4) SMA 2 (22) SMA 3 (12)	10 months	↑IL-10, ↑leukocyte counts, ↑MCP-1/CCL2	No association
[76]	In serum extracellular vesicles: *SMN* transcript In serum and CSF: pNfH	Serum, CSF, serum extracellular vesicles		SMA 2	14 months	↑*flSMN* transcript, ↓pNfH	
[82]	Total tau, NfL, S100B	CSF	26	SMA 1 (13) SMA 2 (4) SMA 3 (9)	5–15 doses	↓Total tau	Not analyzed
[91]	NfL, MCP-1, fractalkine/CXCL3, IP-10/CXCL-10, IL-8/CXCL-8, sAPPα, sAPPβ	CSF	13	Older children with SMA (4) Younger children with SMA (9)	36 months	↓NfL, ↓sAPPα, ↓sAPPβ, ↓IL-8 (only in younger patients), ↓IP-10 (only in younger patients), ↑fractalkine	A negative correlation of IL-8, MCP-1, and sAPPα with CHOP-INTEND score
[100]	CSF miRNA signature	CSF	34	SMA 2 (16) SMA 3 (18)	6 months	Lower baseline levels of miR-206 and miR-133 predict clinical response to nusinersen ↑miR-103b and ↓miR-1-3p, miR-133a/b and miR-206 in nusinersen responders	Inverse correlation of miR-206 with the difference in HFMSE score post and pre-treatment with nusinersen
[56]	NfL and pNfH	CSF and serum	39	Adult SMA patients (33) HCs (6)		Unchanged	A negative correlation of serum pNfH levels with RULM score, upper and lower extremity strength, and total strength
[77]	Whole proteome	CSF	44	SMA 1 (12) SMA 2 (9) SMA 3 (6) Asymptomatic individuals (4) HCs (13)	300 days	↓Cathepsin D, ↓pNfH, ↓pNfL	Cathepsin D alters in response to treatment based on CHOP-INTEND-, HFMSE- and HINE2- scores
[51]	41 miRNAs	Serum	22	SMA 1	6 months	↑miR-335-5p, ↑miR-328-3p, ↑miR-423-3p, ↑miR-142- 5p and ↓miR-26b-5p	Several miRNA levels correlated positively with the functional improvement induced by nusinersen treatment (as measured by the CHOP-INTEND and HINE scales)
[101]	Extracellular RNAs and miRNAs	CSF	12	SMA 1 (4) SMA 2 (5) SMA 3 (3)	4–13 doses	↓expression of 48 genes ↑expression of 53 genes ↑miR-132, miR-218, miR-9, miR-23a, miR-146a	Not analyzed
[83]	pNfH	CSF	11	SMA 2	Up to 34 months	No difference	Not analyzed
[103]	Metabolome	CSF	27	SMA 1 (12) SMA 2 (7) SMA 3 (8)	302 days	-Altered amino acids and glucose metabolism in the CSF of SMA 1 patients -Altered amino acids and ketone body metabolism in the CSF of SMA 2 patients	A negative correlation between 3-hydroxybutyrate levels and CHOP-INTEND scores; a positive correlation between 3-hydroxybutyrate, alanine, and valine and HFMSE scores
[78]	pNfH	Plasma	93	Infantile-onset SMA	Up to 757 days	↓pNfH	Not analyzed
[95]	Total protein, glucose, cell count	CSF	50	SMA 1 (22) SMA 2 (17) SMA 3 (11)	22 months	↑total protein (for SMA 2 and 3)	No association
[48]	Whole proteome	CSF	17	SMA 1 (10) HCs (7)	4 doses (6 months)	↑APOA1 ↑Transthyretin ↓Haptoglobin ↓Hemoglobin sub. β 2D-PAGE analyses showed differential expression of 30 proteins	Not analyzed
[104]	Creatinine kinase, Cystatin C	Serum	16	SMA 3 SMA 4	14 months	No difference	Not analyzed
[45]	In CSF: NfL, total tau, p-tau_181_, Qalb, OCB, CSF white cells In serum: creatinine	CSF, serum	21	SMA 2 (3) SMA 3 (6) HCs (12)	5 (540 days)	↑Qalb, development of systemic and intrathecal OCBs (in some SMA patients)	A positive relationship between creatinine and HFMSE score and RULM
[96]	Leukocyte count, lactate, total protein, Qalb, OCB	CSF	28	SMA 2 (10)SMA 3 (17) SMA 4 (1)	22 months	↑total proteins ↑Qalb	No association with HFMSE, 6MWT, and RULM scores
[58]	CHIT1, total proteins, Qalb	CSF	109	SMA 1 (7) SMA 2 (33) SMA 3 (39) HCs (30)	6 doses (14 months)	↑CHIT1 ↑Qalb ↑total proteins	No association with HFMSE and CHOP-INTEND scores
[90]	Amyloid β_1–40_ and amyloid β_1–42_	CSF	8	SMA 2 (3) SMA 3 (5)	420 days	↑amyloid β_1–42_	Not analyzed
[79]	pNfH, NfL, total tau, neurogranin, BACE-1, α-synuclein	CSF	44	SMA 1 (16) SMA 2 (16) SMA 3 (12)	300 days	↓pNfH (in SMA 1 and 2) ↓NfL (in SMA 1) ↓total tau (in SMA 1 and 2) ↑neurogranin (in SMA 3) ↑α-synuclein (in SMA 1)	Inverse correlation between total tau and pNfH levels and the CHOP-INTEND score in SMA type 1 patients; a positive association between neurogranin and RULM score in SMA type 2 and 3 patients; an inverse correlation of NfL with HFMSE score and pNfH with HFMSE and RULM score (in SMA 2 and 3 patients).
[97]	In CSF: WBC, glucose, lactate, total protein In blood: WBC, platelets, INR, aPTT, Crea, Urea, AST, ALT, GGT	CSF and blood	50	SMA 2 (14) SMA 3 (36)	Up to 12 doses (34 months)	In CSF: ↓Glucose, ↑total protein In blood: ↓aPTT, ↓Crea	Not analyzed
[39]	NfL in CSF and serum	CSF and serum	115	SMA 1 (4) SMA 2 (7) SMA 3 (3) SMA 2–3 (4) HCs (97)	Up to 12 doses (34 months)	↓NfL in CSF and serum	Negative correlation between serum NfL and CHOP-INTEND score
[80]	NfL	CSF	1	SMA 1	12 months	↓NfL	Not analyzed
[60]	NfL	Serum	106	SMA 2 (20) SMA 3 (26) SMA 2/3 (46) HCs (14)	7 doses (over 14 months of treatment)	No difference	Higher levels of NfL are associated with poorer motor performance (as measured using the HFMSE and ALSFRS-R); no correlation between NfL levels and motor scale scores
[98]	Whole proteome, total proteins, Qalb, QIgA, QIgG, QIgM, OCB, glucose, lactate, cell count, reactive mononuclear transformations	CSF	20	SMA 2 (1) SMA 3 (9) HCs (10)	10 months	↑total proteins, ↑Qalb; Two protein clusters were identified after nusinersen treatment that differed from protein clusters at the baseline	After treatment with nusinersen, two protein clusters with substantially different HFMSE scores were identified
[81]	NfL, GFAP	CSF	17	SMA 2 (6) SMA 3 (11)	14.33 months	↓NfL, ↓GFAP (only in SMA 3 patients)	No association with the RULM scores
[102]	Muscle-specific miRNAs (myomiRs); miR-133a, miR-133b, miR-206, miR-1	Plasma	21	SMA 2 (16) SMA 3 (5)	4 doses (6 months)	↓miR-133a ↓miR-133b ↓miR-1	A negative correlation of miR-133a with the HFMSE score
[61]	pNfH, NfL	CSF	21	SMA 3 (12) HCs (9)	6 months	↓pNfH, ↓NfL	No association with the HFMSE score
[62]	NfL, total tau, GFAP	CSF	23	SMA 1 (12) HCs (11)	8 doses	↓NfL, ↓total tau, ↓GFAP	A negative relationship between NfL and total tau levels and the CHOP-INTEND score
[86]	NfH, total tau, S100B and NSE	CSF and serum	11	SMA 3	4 doses	Unchanged	Not analyzed
[99]	White cell count, total protein, Qalb, lactate, and OCB	CSF	60	SMA 1 (2) SMA 2 (28) SMA 3 (30)	540 days	↑total protein, ↑Qalb	No association with HFMSE score
[84]	pNfH, NSE, amyloid β_1–40_, amyloid β_1–42_, p-tau, tau protein, proteins, creatine kinase	CSF	19	SMA 3	300 days	↓NSE, ↓p-tau	Not analyzed
[63]	pNfH	Plasma	155	SMA 1 (121, of whom 48 completed treatment) HCs (34)	5 doses (302 days)	↓pNfH	A negative association between pNfH and CHOP-INTEND score
[105]	SMN protein	CSF	28	SMA 2 (15) SMA 3 (13)	9–14 months	↑SMN protein	Correlation between SMN protein and enhancement in motor function (as measured using HFMSE score)

(↑) increase, (↓) decrease. The literature search was completed on 2 May 2023. 6MWT, 6-min walk test; ALSFRS-R, Amyotrophic Lateral Sclerosis Functional Rating Scale-Revised; ALT, alanine aminotransferase; ANG1, angiopoietin-1; APP, amyloid precursor protein; aPTT, activated partial thromboplastin time; AST, aspartate aminotransferase; BDNF, brain-derived neurotrophic factor; C5a, complement C5 alpha chain; crea, creatinine; CHIT1, chitotriosidase 1; CHOP-INTEND, The Children’s Hospital of Philadelphia Infant Test of Neuromuscular Disorders; CSF, cerebrospinal fluid; FGF, fibroblast growth factor; flSMN, full-length SMN; G-CSF, granulocyte-colony stimulating factor; GFAP, Glial fibrillary acidic protein; GGT, gamma-glutamyltransferase; GM-CSF, Granulocyte macrophage colony stimulating factor; HC, healthy control; HFMSE, Hammersmith Functional Motor Scale Expanded; HINE, Hammersmith Infant Neurologic Examination; HINE2, HINE, Section 2; IFN, Interferon; IL, interleukin; INR, international normalized ratio; IP-10, interferon gamma-induced protein 10; MCAF/MCP-1, monocyte chemoattractant protein 1; MCP-1, monocyte chemoattractant protein 1; MIP, Macrophage inflammatory protein; miRNA, micro RNA; NfH, neurofilament heavy chain; NfL, neurofilament light chain; NSE, neuron-specific enolase; OCB, oligoclonal immunoglobulin G (IgG) bands; pNfH, phosphorylated neurofilament heavy chain; pNfL, phosphorylated neurofilament light chain; PDGF-BB, platelet-derived growth factor-BB; p-tau, phosphorylated tau protein; p-tau_181_, tau protein phosphorylated at threonine 181; Qalb, CSF/serum quotient of albumin; QIgA, CSF/serum quotient of IgA; QIgG, CSF/serum quotient of IgG; QIgM, CSF/serum quotient of IgM; RULM score, Revised Upper Limb Module score; S100B, S100 calcium-binding protein B; SMA, spinal muscular atrophy; SMN, survival motor neuron; TNF-α, tumor necrosis factor alpha; urea, urea nitrogen; VEGF, vascular endothelial growth factor; WBC, white blood cell count.

### 5.3. Limitations and Future Perspectives

The main limitations for the usage of molecular biomarkers in clinical practice are: (1) a lack of general agreement and recommendations between different countries for the best molecular biomarker choice; (2) a lack of agreement and recommendations for body fluids in which biomarkers should be analyzed (CSF vs. serum or plasma); (3) inconsistencies in the results between different studies due to the usage of different analytical methods or analysis of different bodily fluids; (4) high cost of molecular biomarker analysis. The debate is still ongoing regarding whether molecular biomarkers should be prioritized between imaging and electrophysiological biomarkers when monitoring disease progression and therapeutic response (since genetic biomarkers cannot be used to monitor therapeutic response). With the main purpose of addressing these issues, the recently formed SMA Multidisciplinary Biomarkers Working Group consists of 11 experts in the field of SMA research [106]. The main goal of this Working Group is to provide recommendations for the usage of prognostic, predictive, and pharmacodynamic biomarkers of SMA in clinical practice [106]. Several biomarkers have been considered: (1) biomolecular biomarkers (Nf, SMN protein, and muscle indicators [creatinine, creatine kinase, and markers of muscle damage]); (2) genetic biomarkers (copy number or polymorphisms of the *SMN2* gene and the expression of modifier genes); (3) gene transcription and splicing regulators (miRNAs, long non-coding RNAs, methylation factors); (4) imaging biomarkers (EIM and muscle imaging using MRI); and (5) electrophysiological biomarkers (repetitive nerve stimulation (RNS), CMAP, and MUNE). The top biomarker among the aforementioned biomarkers was Nf, and the Working Group recommended Nf for further research and development since it showed prognostic, predictive, and pharmacodynamic potential [106].

Studies like the one performed by Glascock et al. [106] are very important given the approval of three disease-modifying therapies for SMA and an increase in newborn screening for SMA.

## 6. Conclusions

To date, numerous genetic, molecular, imaging, and electrophysiological biomarkers have been evaluated to identify diagnostic, prognostic, and pharmacodynamic SMA biomarkers. In this review, we examined the diagnostic, prognostic, and pharmacodynamic potential of molecular markers measured in the biological fluids of patients with SMA. The *SMN2* copy number is the most accurate genetic biomarker in the natural course of the disease, although it cannot be used as a pharmacodynamic biomarker because it remains constant throughout an individual’s lifetime. However, the *SMN2* copy number can be used in the choice of proper treatment. For example, onasemnogen abeparvovec is approved for the treatment of children with SMA type 1 and presymptomatic individuals carrying two or three copies of the *SMN2* gene [107]. Recent reviews [37,108] concluded that SMN-related biomarkers (either mRNA- or protein-based) are unreliable predictors of disease severity. NfL and pNfH, on the other hand, demonstrated positive results in disease severity prediction and therapeutic response monitoring. Numerous additional biomarkers were also evaluated as prognostic and pharmacodynamic SMA biomarkers (Table 1 and Table 2). Several investigations have analyzed the entire proteome, metabolome, transcriptome, and miRNAome to identify new potential SMA biomarkers. However, it is essential to keep in mind that SMA is a complex disease with numerous subtypes. This could contribute significantly to the disparities among the studies. Moreover, a recent study has convincingly shown that t-tau levels decrease significantly in patients with SMA 1 (but not SMA 2 and 3) when treated with nusinersen [82]. More research is needed to discover which biomarker (or combination of biomarkers) is best for predicting disease severity, evaluating therapeutic response and separating treatment responders from nonresponders.

## Data Availability

Data are available from the corresponding author upon reasonable request.

## Abbreviations

2D-PAGE, two-dimensional SDS-polyacrylamide gel electrophoresis; ALS, Amyotrophic Lateral Sclerosis; ALSFRS-R, Amyotrophic Lateral Sclerosis Functional Rating Scale-Revised; ALT, alanine aminotransferase; ANG1, angiopoietin-1; APP, amyloid precursor protein; aPTT, activated partial thromboplastin time; AST, aspartate aminotransferase; BDNF, brain-derived neurotrophic factor; BSID-III, Bayley Scales of Infant and Toddler Development, third edition; C5α, complement C5 α chain; crea, creatinine; CHIT1, chitotriosidase 1; CHOP-INTEND, The Children’s Hospital of Philadelphia Infant Test of Neuromuscular Disorders; CMAP, compound muscle action potential; CORO1C, coronin 1C; CSF, cerebrospinal fluid; DMD, Duchenne muscular dystrophy; ECL, electrochemiluminescence; EIM, electrical impedance myography; ELISA, enzyme-linked immunosorbent assay; FDA, Food and Drug Administration; FGF, fibroblast growth factor; flSMN, full-length SMN; FL, Full Length; G-CSF, granulocyte-colony stimulating factor; GFAP, glial fibrillary acidic protein; GGT, gamma-glutamyltransferase; GM-CSF, Granulocyte macrophage colony stimulating factor; HC, healthy control; HFMS, Hammersmith Functional Motor Scale; HFMSE, Hammersmith Functional Motor Scale Expanded; HINE, Hammersmith Infant Neurologic Examination; HINE2, HINE, Section 2; HSPA7, Heat Shock Protein Family A Member 7; HSP70B, heat shock 70 kDa protein 7; IFN, Interferon; IL, interleukin; INR, international normalized ratio; IP-10, interferon γ-induced protein 10; MCAF/MCP-1, monocyte chemoattractant protein 1; MCP-1, monocyte chemoattractant protein 1; MHFMS, Modified Hammersmith Functional Motor Scale; MIP, Macrophage inflammatory protein; miRNA, microRNA; MRI, magnetic resonance imaging; MS, MALDI-TOF mass spectrometry; MFM, Muscle function measurement; MRC, Medical research council; MUNE, motor unit number estimation; NAIP, NLR family apoptosis inhibitory protein; Nf, neurofilament; NfH, neurofilament heavy chain; NfL, neurofilament light chain; NfM, neurofilament medium chain; NMR, nuclear magnetic resonance; NSE, neuron-specific enolase; OCB, oligoclonal immunoglobulin G (IgG) bands; PBMC, peripheral blood mononuclear cells; PDGF-BB, platelet-derived growth factor-BB; p-tau, phosphorylated tau protein; pNfH, phosphorylated neurofilament heavy chain; pNfL, phosphorylated neurofilament light chain; p-tau_181_, tau protein phosphorylated at threonine 181; Qalb, CSF/serum quotient of albumin; QIgA, CSF/serum quotient of IgA; QIgG, CSF/serum quotient of IgG; QIgM, CSF/serum quotient of IgM; RNS, repetitive nerve stimulation; RULM score, Revised Upper Limb Module score; S100B, S100 calcium-binding protein B; SMA, spinal muscular atrophy; SMN, survival motor neuron; TIMPSI, Test of Infant Motor Performance Screening Items; TNF-α, tumor necrosis factor α; urea, urea nitrogen; VEGF, vascular endothelial growth factor; WBC, white blood cell count; WB, Western blot.
